# Production of recombinant proteins including the B-cell epitopes of autolysin A of *Staphylococcus aureus* isolated from clinical sheep mastitis and their potential for vaccine development

**DOI:** 10.1007/s11259-023-10121-1

**Published:** 2023-04-19

**Authors:** Elisa Azara, Antonio Carlo Foddai, Carla Maria Longheu, Maria Filippa Addis, Sebastiana Tola

**Affiliations:** 1 Istituto Zooprofilattico Sperimentale della Sardegna “G. Pegreffi”, Sassari, 07100 Italy; 2 School of Biological Sciences, University of Aberdeen, Aberdeen, Scotland; 3 Dipartimento di Medicina Veterinaria, Università degli Studi di Milano, Lodi, 26900 Italy; 4 Laboratorio di Malattie Infettive degli Animali (MiLab), Università degli Studi di Milano, Lodi, 26900 Italy

**Keywords:** *Staphylococcus aureus*, Sheep, AtlA, 3D protein structure, B-cell epitope prediction, Recombinant protein

## Abstract

*Staphylococcus aureus* is the most common clinical mastitis-associated pathogen in sheep which contributes to reduced welfare of affected animals and, therefore, compromises the quality and quantity of milk production. To prevent mastitis and its spread, it is essential to guarantee adequate breeding conditions and animal health, through the adoption of good farm management practices and the application of suitable biosecurity measures. Vaccination can play a strategic role in prevention, control, and eradication of diseases. The identification of secreted and cellular antigens of the predominant sheep-CC130/ST700/t1773 lineage would assist in the design of effective vaccine against mammary infections caused by *S. aureus.* In the current study, we carried out a 3D structural prediction analysis with the identification of the best B cell epitopes of the whole and secreted portion of *S. aureus* AtlA. Fragments of *atl*A, containing the main predicted epitopes, were amplified, cloned, and expressed in *Escherichia coli* for recombinant protein production. Two selected clones produced recombinant proteins (rAtl4 and rAtl8) showing strong reactivity with a hyperimmune serum against the native AtlA and with blood sera collected from sheep with clinical *S. aureus* mastitis. These may represent potential candidate protein-based vaccines able to elicit a protective immune response to be evaluated by vaccination and subsequent challenge of the vaccinated sheep.

## Introduction

*Staphylococcus aureus* is a major pathogen of dairy small ruminants, responsible for most of clinical intramammary infection (IMI) cases (Marogna et al. 2010; Dore et al. 2016). Along with the application of adequate flock management procedures including hygiene, biosafety measures, proper milking and equipment maintenance, regular monitoring of animals, treatment and or elimination of positive ewes, a strategic role in controlling *S. aureus* IMI can also be played by vaccination, as recommended by the guidelines for the prudent use of antimicrobials in veterinary medicine (2015/C 299/07). In Italy, two commercial vaccines are currently available for immunisation of small ruminants against *S. aureus* IMI. These are based on inactivated *S. aureus* strains expressing biofilm components alone or combined with alpha and beta haemolysins.

The Istituto Zooprofilattico della Sardegna (IZSSA) is authorized by Italian Health Ministry to produce an inactivated autogenous vaccine based on the *S. aureus* isolate involved in the outbreak, with the aim of limiting infection spread within the flock. However, vaccines based on epitopes are preferred to whole antigens for eliciting a specific immune response against viral and bacterial pathogens (Soria-Guerra et al. 2015). The production of an effective vaccine is subordinated to the knowledge of the natural *S. aureus* population circulating in the geographic area, since local strains may have evolved region-specific pathogenicity characteristics. In previous studies, we observed that the prevalent *S. aureus* lineage associated with ewe mastitis in Sardinia is CC130/ST700/t1773, a very ancestral lineage dating over five thousand years ago (Azara et al. 2017a). Our studies also demonstrated the absence of biofilm production traits in all the isolates analysed so far (Azara et al. 2017b). The absence of biofilm production genes and the presence of beta-haemolysin genes only in 44% of Sardinian isolates led us to search for alternative proteins. To this aim, we investigated the antibody response elicited in sheep with clinical mastitis by *S. aureus* (Longheu et al. 2020). In that study, we identified a total of 7 immunogens by immunoproteomics, including the extracellular protein autolysin A (AtlA).

AtlA is a well-known cell-surface associated multifunctional protein (Biswas et al. 2006) involved in cleaving the peptidoglycan (PG) layer and in cell separation during bacterial replication (Zoll et al. 2010). Specifically, AtlA includes two domains with hydrolytic activity, an amidase (AM) and a glucosaminidase (GM), that cleave the PG at different locations (Buttner et al. 2014). The active 62 kDa AM and 51 kDa GM proteins are generated by post-translational processing of the full-length precursor (Schlag et al. 2010). AM is the major contributor in staphylococcal pathogenesis as it binds various extracellular proteins such as fibronectin, heparin, and gelatin, thus facilitating colonization and infection (Porayath et al. 2018). Since cellular adhesion is the first step of bacterial invasion, the immunogenic AtlA protein represents a suitable candidate for sheep vaccination. In addition, the choice of this protein is strengthened by the fact that almost all (97.3%) biofilm-negative *S. aureus* isolates were PCR positive for *atl* gene (Azara et al. 2017b).

With these premises, we carried out protein structure and B-cell epitope prediction of the secreted portion of *S. aureus* AtlA and finally produced recombinant proteins including all the predicted epitopes of interest. These were then tested for reactivity with a hyperimmune serum against the native AtlA and then with blood sera collected from sheep with clinical *S. aureus* mastitis.

## Materials and methods

### *S. aureus* culture and antigen preparation

Twenty field sheep *S. aureus* isolates were randomly selected from our collection to verify the presence of AtlA in both pellet and culture supernatants. Isolates were grown in 5 ml of Brain Heart Infusion broth (BHI, Oxoid LTD, Basingstoke, UK) at 37 °C for 18 h with shaking. Overnight culture was pelleted by centrifugation at 1945 X g for 10 min. The pellet was resuspended in phosphate buffered saline (PBS) at the final concentration of one-tenth of the original volume while the supernatant was concentrated using Amicon® Ultra spin columns (Sigma-Aldrich, St. Louis, MO, USA) at 3800 X g for 20 min. The presence of secreted AtlA protein in the pellet and supernatant was evaluated by immunoblotting using a hyperimmune anti-secreted AtlA serum produced in lamb, according to the procedure described by Longheu et al. (2020).

### Determination of *S. aureus* AtlA structure

The complete sequence of AtlA (1257 amino acids) was retrieved via the NCBI protein database (https://www.ncbi.nlm.nih.gov) as FASTA format. Protein structure prediction of AtlA (i.e. 1257 aa and 577 aa portions) based on the crystal structure of the homologous protein bifunctional autolysin from the *S. aureus* strain Mu50/ATCC 700,699 (PDB entry 6fxo) was carried out in July 2020 using two online search tools: I-TASSER (Yang et al. 2015) and In-Fold 5 (McGuffin et al. 2019). All the residues differing between the two protein sequences were then removed from each preliminary search model using CHAINSAW (Schwarzenbacher et al. 2004). Amended protein models were automatically created using BUCCANEER (Cowtan 2006). Additional manual rebuilding of the model was undertaken when required using Coot (Emsley and Cowtan 2004). Crystallographic refinement was finally carried out using REFMAC5 (Murshodov et al. 2011).

### B-cell epitope prediction analysis

The following B-cell epitope prediction tools were used: (a) ElliPro (based on a protein antigen’s 3D structure - Protrusion Index method) (Ponomarenko et al. 2008); (b) BcePred (based on exposed surface - Janin method) (Saha and Raghava 2006); (c) BepiPred 2.0 (based on Jespensen method) (Jespersen et al. 2017); (d) IEDB (based on antigenity score-Kolaskar and Tongaonkar method) (Kolaskar and Tongaonkar 1990). The overall accuracy of computational results was deemed based on the level of general agreement observed among the different tools applied in parallel. The epitopes more frequently identified by most of the tools were finally selected (Table 1).

**Table 1:** Results of B-cell epitope prediction analysis carried out on secreted AtlA protein (i.e. portion 215–791) using 4 online available servers: Ellipro (threshold 0.7); BcePred (threshold 0.6); BepiPred 2.0 (threshold 0.6); and Immune Epitope Database Server (IEDB, threshold 0.95)

							Epitope prediction tools									Selected top-scoring epitopes areas				
**Epitopes**	ElliPro					BcePred				BepiPred 2.0			IEDB							
**No**	**Start**	**End**		**Score** ^a^		**Start**		**End**	**Score** ^b^	**Start**	**End**	**Score** ^b^	**Start**	**End**	**Score** ^b^	**Start**	**End**	**Length (aa)**	**Sequence**	
1	1	28		0.86		6		20	0.67	4	8	0.9	4	32	0.99	4	20	16		VNSSINDYIRKNNLKA
2	32	48		0.76		34		46	0.69	24	46	0.65	39	62	0.97	34	46	13		YAYRNGVGRPEGI
3	171	225		0.73		192		230	1.12	181	259	1.37	153	226	1.017	181	225	45		DQLYDLINEKYLIKMGKVAPW GTQSTVAPWGTQSTTTPTTPSKPSTPSKPSTPS
4	399	420		0.75		385		409	1.31	398	417	1.96	380	417	0.99	399	409	22		TVSSLNGVAQINAKNNGLFTTV
5	427	446		0.70		426		442	0.61	409	436	0.60	423	469	1.019	427	436	14		PTKEVQKTFAVTKE
6	469	525		0.78		456^c^		468^c^	0.75	449^c^	475^c^	0.65	473^c^	499^c^	1.03	469	520	52		IYNNAKSPVNVMQTYTVKPGTKLYSVPWGTYKQEAGAVSGTGNQTFKATKQQ
						476^c^		482^c^	0.83	495^c^	520^c^	0.98	505^c^	551^c^	1.02					
						590^c^		517^c^												

^a^ Overall score assigned by ElliPro to each one of the selected top-scoring epitope areas

^b^ Overall score calculated manually as an average of individual scores assigned by BcePred, BepiPred 2.0 and IEDP to each individual residue of the selected top scoring areas

^c^ Multiple discontinuous top-scoring epitope areas identified by BcePred, BepiPred 2.0 and IEDP in the portion 215–791

### DNA extraction, primers design and cloning of selected AtlA fragments

Genomic DNA was extracted from the field sheep *S. aureus* isolate 34,074, according to the protocol described by Onni et al. (2012). The aminoacid sequences and the oligonucleotide primers used for recombinant protein production are indicated in Table 2. The oligonucleotide sets were designed for the directional and ORF cloning of the gene fragments by inserting engineered restriction sites in forward and reverse primers. Both PCR products and the pQE-30 expression vector (Qiagen, Chatsworth, CA, USA) were digested with *Bam*HI and *Kpn*I restriction enzymes. The double-digested pQE-30 plasmid was dephosphorylated with calf intestinal alkaline phosphatase (CIP, Sigma-Aldrich) for 60 min at 37 °C and purified using the Micropure-EZ enzyme removers (Sigma-Aldrich). After determining the ratio of each DNA insert to pQE vector, the ligation was performed by the T4 DNA ligase enzyme (Thermo Scientific, Vilnius, LT) at 25 °C for 1 h. Each construct was used to transform *Escherichia* (*E.*) *coli* DH5α cells containing pREP-4 repressor plasmid (*lac*Iq). Cloning into pQE-30 makes it possible to produce recombinant proteins linked to a polyhistidine (His) stretch that binds strongly to nichel-chelated columns (Qiagen). pQE-30 derivatives were selected in LB agar plates supplemented under with 100 µg/ml ampicillin and 50 µg/ml kanamycin. Plasmid DNA was extracted with the Qiagen Plasmid mini kit (Qiagen).

**Table 2:** Primer sequences, primer combinations and PCR cycling conditions used to amplify *S. aureus Atl*A gene

Aminoacid sequence		Nucleotide sequence (5’-3’)	Reference
1) EATPKV		*Bam*HI-GAAGCGACACCTAAAGTA	This study
2) THYAVS		*Bam*HI-ACTCACTACGCTGTAAGT	This study
3) TPTPKP		*Bam*HI-ACACCAACACCTAAGCCA	This study
4) RSTING		*Kpn*I-ACCATTTATCGTCGAACG	This study
5) NNGVAQ		*Kpn*I-TTGTGCGACACCATTGTT	This study
6) LAVPAA		*Kpn*I-TGCAGCAGGTACAGCTAA	This study
		Combinations	
Primers		Amplification condition	Amplicon size
1 + 4	*Initial denaturation: 94 °C x 5 min; final extension:72 °C x 10 min* *30 cycles 94 °C x 40 s; 50 °C x 40 s, 72 °C x 40 s.*		240 bp
1 + 5	*Initial denaturation: 94 °C x 5 min; final extension:72 °C x 10 min* *30 cycles 94 °C x 60 s; 50 °C x 60 s, 72 °C x 90 s.*		753 bp
1 + 6	*Initial denaturation: 94 °C x 5 min; final extension:72 °C x 10 min* *30 cycles 94 °C x 60 s; 58 °C x 60 s, 72 °C x 120 s.*		1050 bp
2 + 5	*Initial denaturation: 94 °C x 5 min; final extension:72 °C x 10 min* *30 cycles 94 °C x 40 s; 50 °C x 40 s, 72 °C x 40 s.*		261 bp
3 + 6	*Initial denaturation: 94 °C x 5 min; final extension:72 °C x 10 min* *30 cycles 94 °C x 60 s; 52 °C x 60 s, 72 °C x 60 s.*		492 bp

### Expression and purification of recombinant proteins

*E. coli* transformants containing the expected inserts were grown in LB medium until log phase and then induced with 1 mM isopropyl-thio-β-d-galactoside (IPTG) at 37 °C for 1, 3 and 18 h. After centrifugation at 4000 X g for 15 min, pellets were resuspended in PBS and analysed by SDS-PAGE and immunoblotting using anti-His serum (Qiagen), pooled serum from naturally *S. aureus* infected sheep (Longheu et al. 2020) and anti-AtlA hyperimmune serum, as described below. The His-tagged proteins were extracted from selected recombinant clones using the buffer B (8 M urea, 0.1 M NaH2PO4, 0.01 M Tris-HCl, pH 8.0) at room temperature for 1 h under continuous shaking agitation and then purified with the Ni-NTA Spin kit (Qiagen), according to the manufacturer’s instructions.

### SDS-PAGE and immunoblotting (IB)

Whole-cell antigens from selected recombinant *E. coli* and each His-tagged protein were electrophoresed on 12% (w/v) polyacrylamide gels. The apparent molecular mass of recombinant proteins was determined using markers (kaleidoscope pre-stained standards, Bio-Rad, Hercules, CA, USA). Electrophoresed proteins were transferred to nitrocellulose membranes in a Trans-Blot-semidry-apparatus (Bio-Rad), as described by the manufacturer. Blots were incubated for 1 h at 37 °C with anti-His serum diluted 1:500, sheep sera diluted 1:100 in PBS-2% skim milk and anti-AtlA serum diluted 1:500. After several washings with phosphate-buffered saline with 2% skim milk, blots were incubated for 1 h at 37 °C with alkaline phosphatase-conjugated anti-mouse IgG (Sigma-Aldrich) diluted 1:30.000, or alkaline phosphatase-conjugated anti-sheep antibody (Sigma-Aldrich) diluted 1:10.000. After three more washes, blots were developed with bromochloroindolyl phosphate/nitroblue tetrazolium (BCIP/NBT, Promega, Madison, WI) in alkaline phosphatase buffer (100 mM NaCl, 5 mM MgCl2, 100 mM Tris, pH 9.5).

## Results

### Detection of secreted AtlA protein in sheep *S. aureus* isolates

**Fig. 1:**
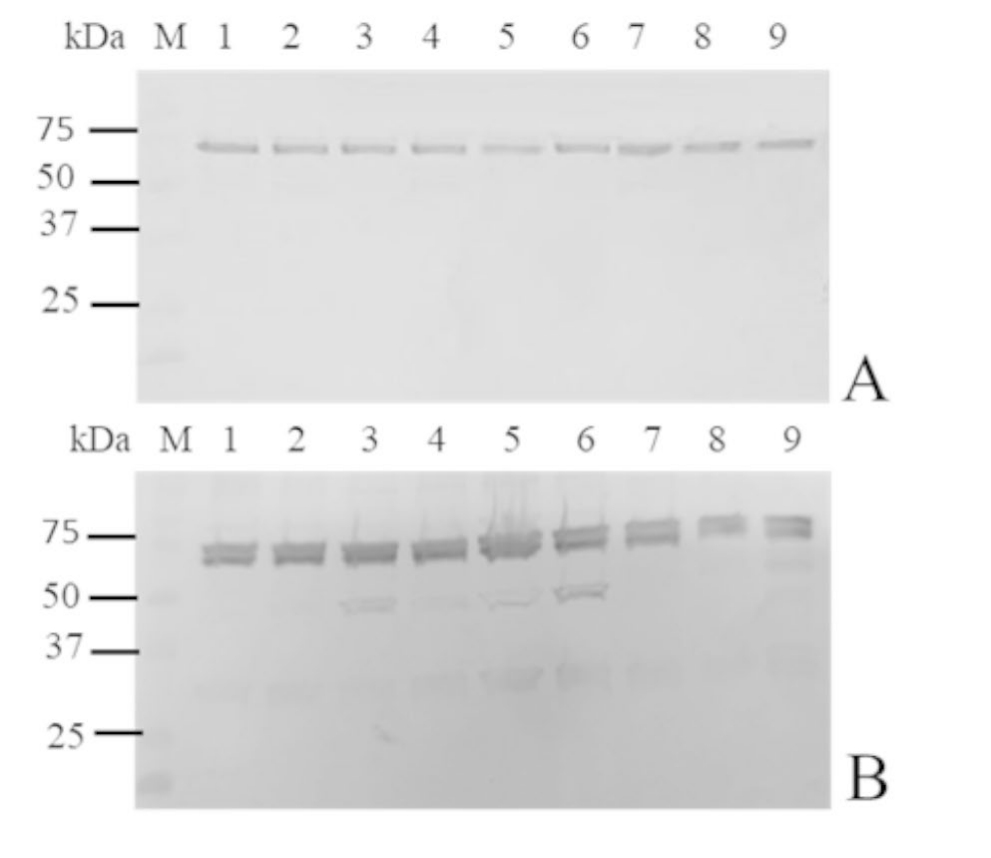
Western Immunoblotting reactivity of anti-AtlA antibodies on the secreted (panel A) and cellular proteins (panel B) of field S. aureus isolates. Immune reactivity was observed on isolates 2089 (lane 1), 1495 (lane 2), 2620 (lane 3), 9625 (lane 4), 4902 (lane 5), 4992 (lane 6), 4460 (lane 7), 3817 (lane 8), and 34,074 (lane 9). Lane M, kaleidoscope protein standards (Bio-Rad).

The anti-secreted AtlA serum was tested against the concentrated supernatant and cellular proteins obtained from twenty sheep *S. aureus* field isolates. As shown in Fig. 1, the hyperimmune serum detected the secreted-AtlA in all field isolates, both in the cellular pellet and in the secreted protein fractions.

### Protein modelling and epitope prediction analysis of AtlA

**Fig. 2:**
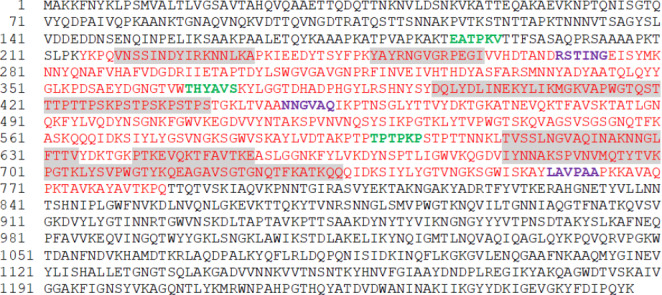
Amino acid sequence of full-length (in black) and secreted portion (in red) of AtlA (NCBI protein, Accession number: WP_001074519.1). Predicted epitopes are highlighted in grey. Forward and reverse primers were designed into the bold green and purple areas, respectively. Primer sequences are detailed in Table 2

The complete sequence of Atl (1257 amino acids) available in the NCBI protein database (https://www.ncbi.nlm.nih.gov) and used for this study is reported in Fig. 2, with the previously sequenced 577 aminoacid secreted portion (Longheu et al. 2020) indicated in red. Several algorithms based on different physico-chemical properties including hydrophilicity, flexibility, accessibility, polarity, and exposed surface have increasingly become available over the last decade for predicting the continuous and discontinuous B-cell epitopes (Soria-Guerra et al. 2015). Accordingly, we first used available tools to predict AtlA protein models for both 1257 aa and 577 aa portions based on the crystal structure of the homologous protein bifunctional autolysin from the *S. aureus* strain Mu50/ATCC 700,699 (PDB entry 6fxo). Linear B-cell prediction analysis was then carried out using 4 individual tools such as Ellipro, BcePred, BepiPred2 and IEDB applied in parallel (see material and methods). Predicted epitopes are indicated in grey in Fig. 2 and detailed in Table 1.

### General features of *S. aureus* AtlA and position of epitopes

**Fig. 3:**
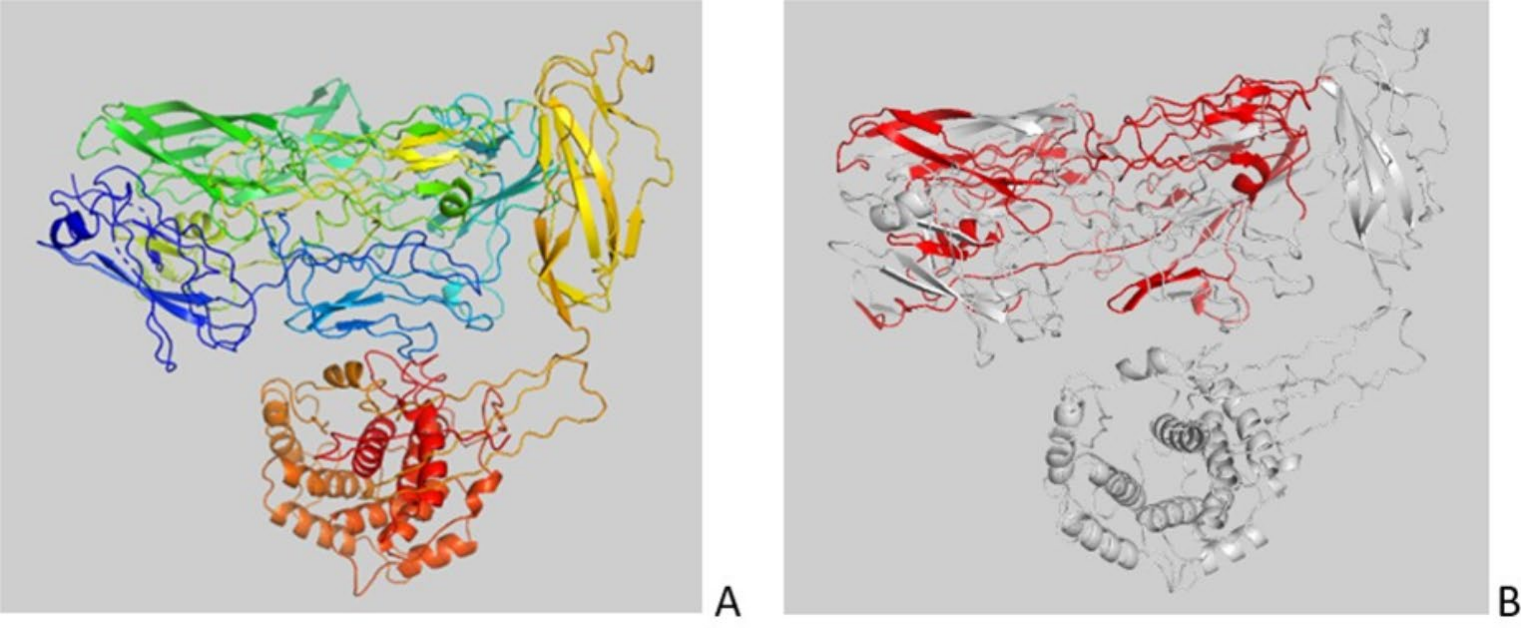
Ribbon diagram representation of Atl protein complete sequence 1257 aa depicted using PyMOL version 2.5.1 (Schrödinger Inc, USA). (**A**) Protein structure is coloured using a rainbow colour scheme starting from blue for the N-terminal portion then gradually switching to red as approaching the C-terminal extremity of the molecule. (**B**) Spatial position of secreted AtlA protein (577 aa) in the crystal structure of Atl protein complete sequence (1257aa). Sequenced portions of the secreted AtlA protein (577 aa) are represented in red. Remaining portions of Atl protein complete sequence (1257 aa) are coloured in grey

**Fig. 4:**
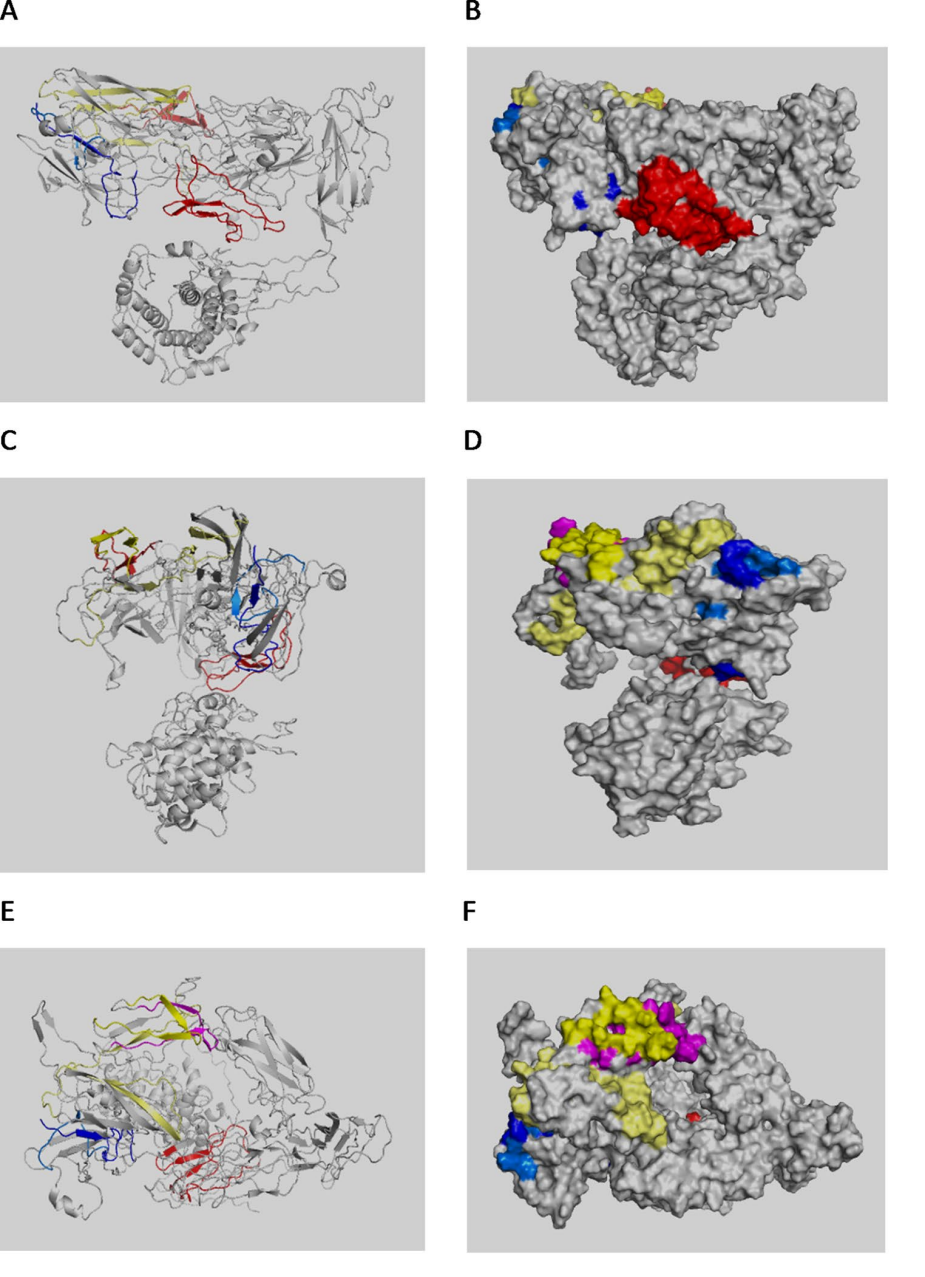
Ribbon diagram (**A, C, E**) and orthographic molecular surface representation (**B, D, F**) of Atl protein complete sequence (1257 aa) and spatial position of selected top scoring epitopes. A and B show the right side of the protein; C and D represent a view from the back obtained by rotating the protein anti-clockwise by 90 degrees; E and F show a view from the top. Not relevant epitopes were identified in the front of the protein (i.e. not included in this section). Epitope areas represented in the three sides of the protein include: Atl4-20 (dark blue); Atl34-46 (light blue); Atl181-225 (red); Atl399-409 (magenta); Atl427-436 (yellow) and Atl469-520 (pale yellow)

The *S. aureus* AtlA protein (Accession number: WP_001074519.1) consists of 1257 a.a. with a molecular weight of 137.44 kDa. The secreted protein (577 a.a.), containing the N-acetylmuramoyl-L-alanine-amidase (314 a.a.) fragment, has a molecular mass of about 64 kDa. The three-dimensional (3D) structure of whole and secreted protein is shown in Fig. 3, while the spatial position of selected top scoring epitopes is shown in Fig. 4.

### Recombinant protein production

**Fig. 5:**
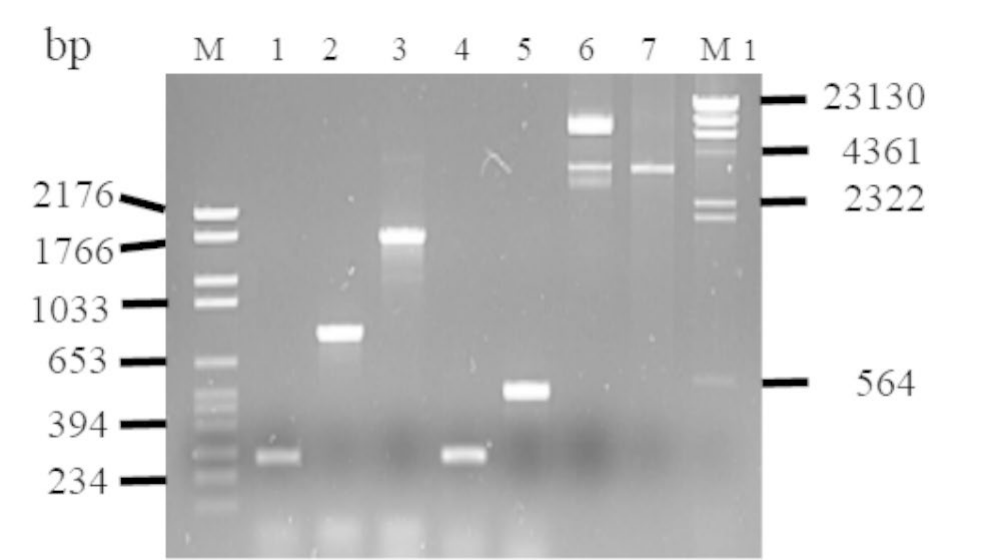
Agarose electrophoresis of PCR products from the *Atl* gene of *S aureus* isolate 34,074. Amplicon sizes were listed in the Table 2. Lane 1, amplicon obtained with primers 1 + 4 (240 bp); lane 2, amplicon obtained with primers 1 + 5 (753 bp); lane 3, amplicon obtained with primers 1 + 6 (1050 bp); lane 4, amplicon obtained with primers 2 + 5 (261 bp); lane 5, amplicon obtained with primers 3 + 6 (492 bp), lane 6, undigested pQE; lane 7, pQE digested with *Bam*HI and *Kpn*I enzymes; M, Marker VI (Roche); M1, Marker II (Roche)

Figure 5 shows the five 240, 261, 492, 753 and 1050-bp fragments of *Atl*A gene amplified from genomic *S. aureus* isolate 34,074 DNA and cloned into pQE-30 plasmid inserted in *E. coli* DH5α.

**Fig. 6:**
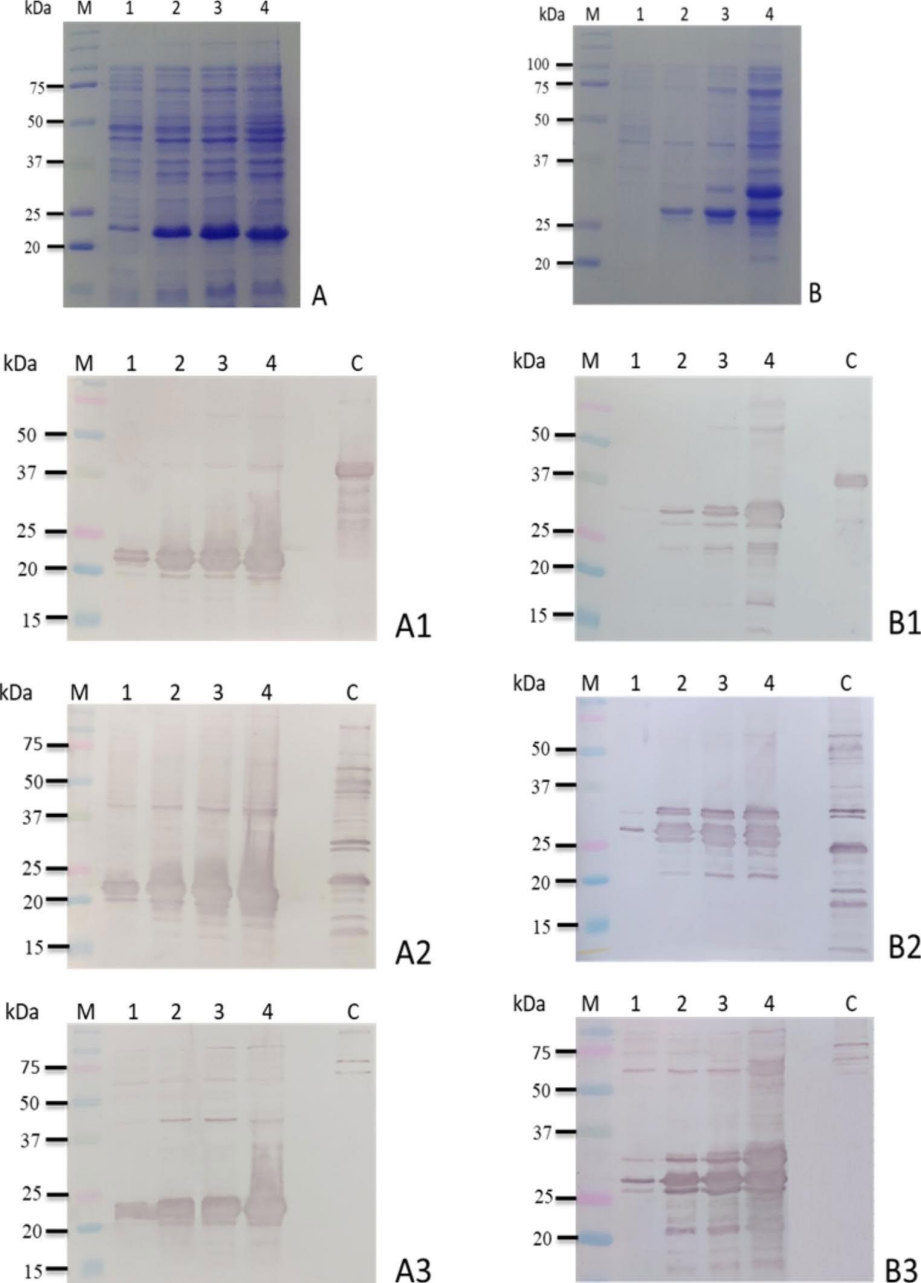
Panels A and B, Coomassie blue-stained SDS-PAGE of the two *E. coli* clones containing the 492-bp (clone 4) and the 753-bp (clone 8) inserts, respectively. Lane 1, *E. coli* culture without IPTG induction; lane 2, *E. coli* culture after 1 h of IPTG induction; lane 3, after 3 h of induction; lane 4, after overnight of IPTG induction. M, kaleidoscope protein standards (Bio-Rad). Panels A1, A2 and A3, IB analysis showing reactivities of anti-His serum (A1), pooled sera from naturally infected sheep (A2) and, anti-AtlA serum (A3) with whole-cell *E.coli* (clone 4) w/o and with IPTG induction. Panels B1, B2 and B3, IB analysis showing reactivities of anti-His serum (B1), pooled sera from naturally infected sheep (B2) and, anti-AtlA serum (B3) with whole-cell *E.coli* (clone 8) w/o and with IPTG induction. Lane C, positive controls: recombinant protein with a poly-His-tag (panels A1- B1), proteins from *S. aureus* supernatant (panels A2- B2 and A3-B3). M, kaleidoscope protein standards (Bio-Rad).

All recombinant *E. coli* clones derivated from the 5-PCR products were analyzed by SDS-PAGE and IB. Only two clones, containing the 492-bp (clone 4) and 753-bp (clone 8) inserts respectively, produced recombinant proteins that reacted with anti-His serum, pooled sheep sera and anti-AtlA serum (Fig. 6).

**Fig. 7:**
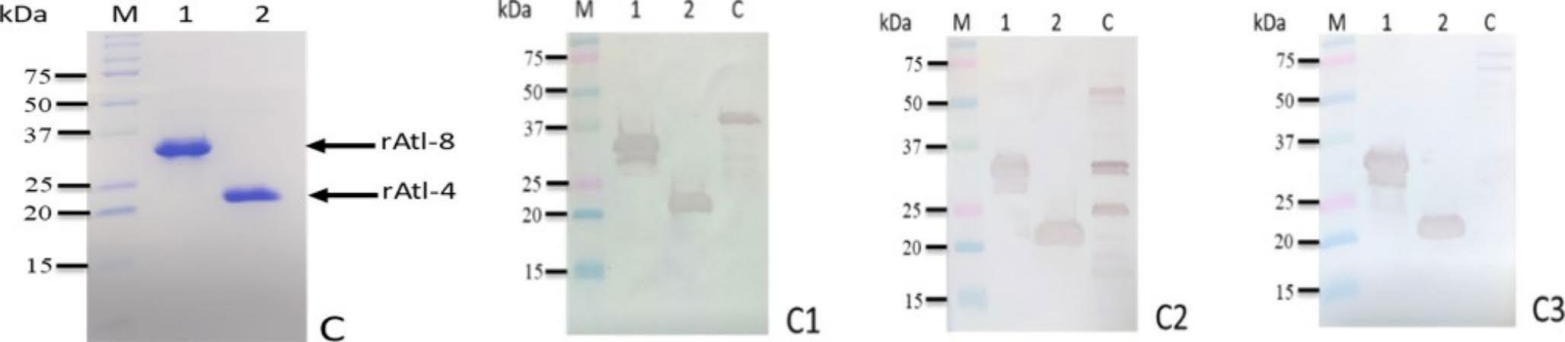
Purification and antigenicity characterization of recombinant AtlA-clone 8 (rAtl-8) and recombinant AtlA-clone 4 (rAtl-4). Panel C, Coomassie blu-stained SDS-PAGE gel with purified rAtl-8 (lane 1) and rAlt-4 (lane 2) from *E. coli* DH5α. Panel C1, Western blotting (WB) results with anti-6His; Panel C2, WB of rAtl-8 and rAlt-4 reactivity with the pooled sera from naturally infected sheep and anti-AtlA serum (panel C3). Lane C, positive controls: recombinant protein with a poly-His-tag (panel C1), proteins from *S. aureus* supernatant (panels C2 and C3). M, kaleidoscope protein standards (Bio-Rad).

The two His-tagged proteins, named rAtl-4 and rAtl-8, presented a molecular mass of approximately 23 and 35 kDa, respectively; more larger than the predicted masses of 18 and 27 kDa. Both recombinant proteins, extracted under denaturing conditions and purified by metal-affinity chromatography, confirm the strong reactivity with the three tested sera (Fig. 7).

## Discussion

The Italian region of Sardinia produces the 68.92% and 57.30% of all Italian sheep and goat milk, respectively. Furthermore, 10% of the sheep milk collected in Europe is Sardinian. The regional production of sheep and goat cheese is estimated at 60,000 tons, of which about 30,000 are Protected Designation of Origin (DOP) cheese (https://www.sardegnaagricoltura.it/index.php?xsl=443&s=413001&v=2&c=6039&vd=1). Mastitis is one of the main causes of the decrease in the efficiency of milk production with consequent repercussions on the dairy industry sector. To prevent mastitis and its spread, and consequently reduce the use of antibiotics, it is essential to guarantee adequate breeding conditions and animal health, placing the emphasis on the adoption of good farm management practices and on the application of adequate biosecurity measures. In the control of mastitis, a strategic role can be played also by vaccination. Vaccines are essential for elicing the immune response and protecting against disease (Vasileiou et al. 2022).

Unfortunately, there is a lack of knowledge regarding the major immunogenic antigens associated with each lineage and which antigens might provide protection against heterologous isolates. In our recent study, we identified the major dominant antigens associated with the CC130/ST700/t1773 *S. aureus* ancestral lineage by an immunoproteomic approach (Longheu et al. 2020). Here, among the secreted and cellular antigens that had functional annotation, we selected the immunogenic AtlA protein, present in almost all the analyzed isolates (Longheu et al. 2020), as a suitable candidate for the development of recombinant proteins to be used for vaccination against *S. aureus* mastitis. Protein-based vaccines have been found to have all the necessary components to initiate T cell-dependent activation of B cells, a process characterized by a more robust immune response, affinity maturation, immunological memory and, simultaneously maintain a good safety profile (Vartak and Sucheck 2016). The bioinformatics approach can perform an appropriate *in silico* selection of epitopes for protein-based vaccine (Saylor et al. 2020). In the present study, computation analysis, one of the most important bioinformatics branches, was used to analyze both the whole and the AM secreted portion of the *S. aureus* AtlA, providing information on the main predicted epitopes and enabling the production of recombinant proteins including these epitopes. Six top-scoring epitope areas within the AM portion were predicted with the highest likelihood of proper epitope presentation, orientation, and exposure. These epitopes were cloned and expressed in *E. coli*, which is one of the earliest and most widespread hosts for the production of heterologous proteins (Terpe 2006). The advantages of this system include ease of cultivation, rapid growth and expression, high product yields and productivity, and low-cost production. It is used for massive production of many commercialized proteins, in particular non-glycosylated proteins (Cid and Bolivar 2021). Post-translational modifications play an important role in protein folding, processing, and stability, as well as biological activity and even the immunogenity/immunoreactivity of the protein (Walsh and Jefferis 2006). To overcome these disadvantages, we only selected the clones that produce recombinant proteins capable of binding the antibodies from naturally infected sheep, anti-AtlA and, anti-His sera.

Of the 5 constructed DNA fragments, only clones 4 and 8, however containing all the 6 epitopes, produced strongly immunoreactive proteins (rAtl4 and rAtl8). Using blood sera collected from sheep with clinical *S. aureus* mastitis (Longheu et al. 2020), we demonstrated that these epitopes are effectively recognized by naturally infected sheep.

Atl mediates adherence of *S. aureus* and exerts peptidoglycal hydrolase activity with associated amidase and glucosaminidase domains. Amidase and glucosaminidase, however, can also bind to host matrices, including fibronectin, thrombospondin 1, vitronectin, and Hsc70, as well as heparin and gelatine (Porayath et al. 2018). Therefore, antibodies against this protein may play an important role in reducing *S. aureus* adhesion, especially when considering that according to our studies biofilm production traits are lacking in all the Sardinian isolates analysed so far (Azara et al. 2017b). Interestingly, it was recently demonstrated that Atl regulates the virulence of *S. aureus* by controlling the sorting of pore-forming leukocidins (Zheng et al. 2022). These toxins mediate leukocyte killing and play a major role in *S. aureus* pathogenesis (Zheng et al. 2021). Previous studies by our group demonstrated that ovine *S. aureus* produce and release significant amounts of lukF-PV/lukM in the extracellular milieu (Longheu et al. 2020), and that the abundance of leukocidin production might be related to the severity of clinical mastitis caused by *S. aureus* in dairy cows (Addis et al. 2022). Therefore, by eliciting the production of neutralizing antibodies, vaccination against Atl might also interfere with this regulatory mechanism, enabling the reduction of *S. aureus* virulence and of clinical mastitis severity. This acquires a particular relevance when considering that most clinical mastitis cases in small ruminants are caused by *S. aureus* (Marogna et al. 2010; Machado 2018). There are mainly three *S. aureus* lineages associated with ovine mastitis: CC133, CC130 e CC522. The first was predominant in Denmark (Eriksson et al. 2013), Netherlands (Hoekstra et al. 2019), Switzerland (Merz et al. 2016); the second in Italy (Azara et al. 2017a), Algeria (Azzi et al. 2020) whereas the third in Spain (Porrero et al. 2012). In dairy cows, vaccination against *S aureus* mastitis has been associated with reduced clinical severity and duration of clinical disease post-challenge (Middleton et al. 2006). Furthermore, reduced transmission within the herd was observed upon vaccination (Schukken et al. 2014). Accordingly, although vaccination strategies may not be completely successful in preventing *S. aureus* infection according to studies carried out in dairy cows (Rainard et al. 2022), these may reduce the incidence and severity of *S. aureus* IMI. When considering that most clinical mastitis cases in sheep are due to *S. aureus*, vaccination may indeed represent a more successful strategy for reducing the impact of mastitis in this dairy animal, in combination with the application of good mastitis prevention protocols, controlling the level of infection in the flock, and monitoring the type of circulating strains.

## Conclusion

Developing a vaccine that can prevent *S. aureus* mastitis in small ruminants is a major challenge. In the current study, we have assessed *in silico* prediction metodologies for identifying the best B cell epitopes within the AtlA protein. In light of the findings obtained with the AtlA protein, further work is being undertaken to extend the computational analysis and the production of recombinant proteins to the other antigens identified in non-biofilm-producing *S. aureus* (Longheu et al. 2020). Then, the ability of candidate protein-based vaccine to elicit a protective immune response could be evaluated by vaccination and subsequent challenge of the vaccinated sheep.

## Data Availability

All data generated or analyzed during this study are includes within the manuscript.
